# The Effects of Lexical Pitch Accent on Infant Word Recognition in Japanese

**DOI:** 10.3389/fpsyg.2017.02354

**Published:** 2018-01-12

**Authors:** Mitsuhiko Ota, Naoto Yamane, Reiko Mazuka

**Affiliations:** ^1^School of Philosophy, Psychology and Language Sciences, University of Edinburgh, Edinburgh, United Kingdom; ^2^Laboratory for Language Development, RIKEN Brain Science Institute, Wako, Japan; ^3^Department of Psychology and Neuroscience, Duke University, Durham, NC, United States

**Keywords:** pitch accent, intonation, Japanese, infants, word recognition

## Abstract

Learners of lexical tone languages (e.g., Mandarin) develop sensitivity to tonal contrasts and recognize pitch-matched, but not pitch-mismatched, familiar words by 11 months. Learners of non-tone languages (e.g., English) also show a tendency to treat pitch patterns as lexically contrastive up to about 18 months. In this study, we examined if this early-developing capacity to lexically encode pitch variations enables infants to acquire a pitch accent system, in which pitch-based lexical contrasts are obscured by the interaction of lexical and non-lexical (i.e., intonational) features. Eighteen 17-month-olds learning Tokyo Japanese were tested on their recognition of familiar words with the expected pitch or the lexically opposite pitch pattern. In early trials, infants were faster in shifting their eyegaze from the distractor object to the target object than in shifting from the target to distractor in the pitch-matched condition. In later trials, however, infants showed faster distractor-to-target than target-to-distractor shifts in both the pitch-matched and pitch-mismatched conditions. We interpret these results to mean that, in a pitch-accent system, the ability to use pitch variations to recognize words is still in a nascent state at 17 months.

## Introduction

### Complexities in Learning Pitch-Based Lexical Contrasts

Infants must learn the sound categories that mark lexical contrasts in their language. Because every language differentiates words using segments (e.g., consonants and vowels), one of the tasks that infants universally have to engage in is to discover segmental phonetic differences that are lexically contrastive. Much of this process takes place during the 1st year and half of life. Infants typically begin to lose perceptual sensitivity to acoustic differences that do not correspond to native segmental categories between 6 and 8 months for vowels (Kuhl et al., 1992; Polka and Werker, 1994) and between 8 and 12 months for consonants (Werker and Tees, 1984). They become able to distinguish familiar and novel words using acoustic differences that do correspond to native segmental categories as early as 11 months (Swingley and Aslin, 2000; Vihman et al., 2004; Swingley, 2005; Mani and Plunkett, 2010).

Some languages also distinguish lexical items with suprasegmental phonetic features such as pitch and duration. There is now a growing body of research on how infants acquire linguistic systems that mark lexical contrasts through variations in pitch, whose primary acoustic correlate is the fundamental frequency (F0) (e.g., Li and Thompson, 1977; Clumeck, 1980; Harrison, 2000; Hua and Dodd, 2000; Mattock and Burnham, 2006; Mattock et al., 2008; Singh et al., 2008; Sato et al., 2010; Singh and Foong, 2012; Yeung et al., 2013; Singh et al., 2015; Singh and Chee, 2016; see Ota, 2016; Singh and Fu, 2016 for overviews). Most previous work on this topic has focused on the development of infants learning a ‘(lexical) tone language,’ a language that specifies the pitch height or contour of the syllables in each word, and comparing that with the development of a language that does not use pitch to mark lexical contrasts (i.e., a ‘non-tone language’).

Findings from this line of research have revealed some interesting characteristics of the developmental trajectories of segmental and tonal contrasts. First, perceptual reorganization for pitch variations appears to occur earlier than that for segmental differences. Infants learning a non-tone language such as English and French lose perceptual sensitivity to certain pitch contrasts (e.g., rising vs. fall-rise) between 4 and 9 months, while infants learning a lexical tone language such as Mandarin, Thai and Yoruba maintain such perceptual sensitivity but also begin to show evidence of native tonal categories as early as 4 months (Harrison, 2000; Mattock and Burnham, 2006; Mattock et al., 2008; Yeung et al., 2013). The onset of these changes precedes the perceptual changes witnessed for segmental contrasts by a few months, suggesting that infants’ ability to adapt to phonetic distributions in the linguistic environment is more advanced for pitch (or F0) than phonetic dimensions related to segments (e.g., voice onset time, formant transitions).

Second, infant learners show robust readiness to incorporate pitch patterns into lexical information, whether or not their language uses pitch to encode lexical contrasts. Perhaps not surprisingly, tone-language learners begin to lexically encode pitch patterns before the end of the 1st year. For example, Singh and Foong (2012) tested Mandarin–English bilinguals on their ability to recognize word forms that were matched or mismatched on the tone of familiarized real words. While 9-month-olds incorrectly recognized both pitch-matched and mismatched Mandarin words, 11-month-olds correctly recognized only pitch-matched words. By 17–18 months, Mandarin-learning infants can also integrate tonal differences in novel word-object associations learned through short laboratory exposures (Singh et al., 2014, 2016). What is unexpected though is that learners of non-tone languages also associate pitch variations with novel word forms, in some cases, up to 18 months (Singh et al., 2014; Hay et al., 2015). In Singh et al. (2014), for example, English-learning 18-month-olds distinguished newly learned words on the basis of pitch patterns. This tendency disappears by 2.5 years, when we see clear evidence that English-learning infants treat pitch-differing words as lexically equivalent, reflecting the non-lexical nature of pitch contrasts in the language (Quam and Swingley, 2010). It should be noted that not all types of pitch contrasts are incorporated into lexical information with equal readiness even when the contrasts are present in the ambient language. In Burnham et al. (2017), both monolingual Mandarin-learning and bilingual English-Mandarin 17-month-olds were able to differentiate novel words on the basis of the native Mandarin high vs. rising tone contrast but not on the native rising vs. falling tone contrast. In addition, bilingual English–Mandarin 17-month-olds were capable of using a non-native (Thai) version of the high vs. rising contrast to learn novel words, but not the non-native Thai rising vs. falling contrast. Thus, infants’ capacity to lexically integrate pitch information is not unique to tone language learners, but it is constrained to some extent by the characteristics of the pitch contrast.

Overall, the existent literature suggests that tonal development is characterized by a precocious perceptual specification for pitch-related contrasts and readiness to incorporate pitch variations as lexical information. However, simple comparison of tone languages and non-tone languages may miss some of the potential complexities involved in mastering pitch phonology. First, the functions played by pitch in human languages are not limited to differentiation of words. In addition to marking lexical contrasts in some languages, pitch variations are also systematically used in intonation (or ‘postlexical’ contrasts) to indicate structures and contrasts above the word level (e.g., phrasal boundaries, focus, question vs. statement) and in paralinguistic expressions to signal speaker states (e.g., emotions, degrees of involvement, arousal) (Ladd, 2008). Because these non-lexical functions of pitch exist in all languages, systematic variations in pitch will be attested even if they are not used to mark lexical contrasts. This can explain why infants learning a non-tone language do not lose their sensitivity to all pitch variations. English-learning infants may become unresponsive to rising vs. low tones, but they continue to show good discrimination of rising vs. falling tones (Mattock et al., 2008), most likely because the latter contrast is encountered in the intonation patterns they are exposed to. It also provides an account as to why learners of non-tone languages remain open-minded about the lexical vs. non-lexical status of pitch as late as 18 months (Singh et al., 2014), as infants must see enough evidence that pitch patterns do not correlate to word-level meanings before they abandon lexical interpretations of tonal variations. The multifunctionality of pitch variations can be a source of challenge to learners of tone languages too, as lexical tones are overlaid on intonational pitch movements. In Mandarin learners, it may not be until 4–5 years of age that children can identify certain tonal differences when they appear in intonational phrases with pitch movements that counteract those of lexical tones (Singh and Chee, 2016). The difficulty exhibited by younger Mandarin learners in learning novel lexical contrasts on the basis of the rising vs. falling contrast compared to the high vs. rising contrast may be attributable to the fact that the rising-falling difference also marks an intonational contrast in the language (Burnham et al., 2017).

A second potential source of complication in learning pitch-based lexical contrasts is that the pitch patterns associated with individual words may not always be constant. Such variability may come from a phonological rule governing lexical tones (i.e., tone sandhi) or an interaction between lexical and intonational features of pitch. An example of tone sandhi is what is known as Sandhi Rule 1 in Mandarin, by which a dipping tone (Tone 3) becomes a rising tone (Tone 2) when followed by another dipping tone. A word like *hen* (‘very’) is therefore produced with either a dipping tone (e.g., *hěn jìn* ‘very near’) or a rising tone (e.g., *hén yuăn* ‘very far’) depending on the following word or morpheme. The variability caused by sandhi may at least partly explain why Mandarin children as old as 3 years of age have difficulty in perceiving and producing the distinction between dipping and rising tones in familiar words (Li and Thompson, 1977; Clumeck, 1980; Wong et al., 2005; Shi et al., 2017). An example of variability introduced by an interaction of lexical and intonational feature can be seen in Swedish. In (Stockholm) Swedish, words fall into two lexical pitch accent categories: Accent 1 and Accent 2. When initially stressed disyllabic words are produced in isolation, Accent 1 words have one pitch peak (e.g., *anden* [scale=.60]img001 ‘the duck’) whereas Accent 2 words have two (e.g., *anden* [scale=.60]img002 ‘the ghost’). However, the second peak in Accent 2 words is an intonational feature (i.e., sentence stress), which disappears in non-focus positions. The variability of word accents caused by the tone-intonation interaction obscures the lexically relevant tonal contrast (Ota, 2006), and may be one of the reasons why Swedish-learning children show confusion between Accents 1 and 2 during their first 2 years (Plunkett and Strömqvist, 1992).

Here we investigate the developmental consequences of these complexities in pitch-based phonology by examining infants’ word recognition in the lexical pitch accent system of Tokyo Japanese. A lexical pitch accent system differs from a canonical tone language system in that tones are specified in words in a much sparser way, usually only on one syllable of the word. But the overall pitch of word is also shaped by intonation, creating a pitch contour that is a composite of lexical and non-lexical features. In a lexical pitch accent system, therefore, the challenge of mastering lexical tone contrasts is compounded by the issues described above. Learners must negotiate, within each word, the components of pitch patterns that are determined by lexical contrasts as opposed to non-lexical factors. They also need to determine how to represent the relevant pitch information that is associated with individual words even when those words may not always carry the same pitch pattern. The details of these aspects of pitch phonology in Japanese are described in the section below.

### Pitch Accent in Tokyo Japanese

**FIGURE 1:**
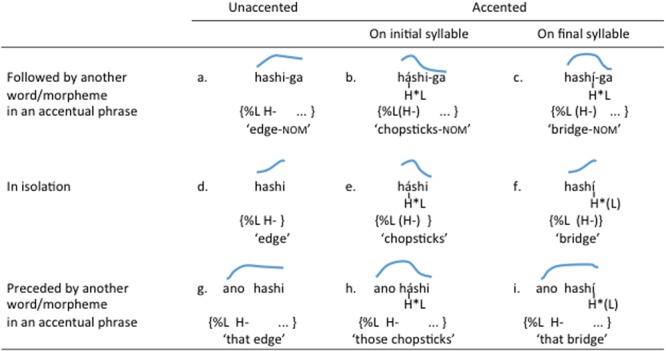
Three segmentally identical Japanese words contrasting in pitch accent. Blue lines are stylized F0 contours. In **(a–c)**, *hashi* is followed by a nominative marker /ga/. In **(d–f)**, it is the only word in an accentual phrase (and therefore, phrase-initial). In **(g–i)**, it is not the initial word in an accentual phrase. Tonal analysis is given below each item. H^∗^L is a pitch accent assigned at the word level. L% marks the onset of the accentual phrase (shown in curly brackets), and is followed by a phrasal H tone (H–).

Tokyo Japanese has only one type of tonal pattern that is lexically relevant, which is realized as a falling pitch contour. Words are either accented or unaccented. Unaccented words are not marked by the lexical falling pitch. Accented words have one ‘accented’ syllable, which carries the falling pitch contour within itself if it contains a long vowel or a nasal coda, but otherwise exhibits the pitch fall between itself and the following syllable. The pitch shape of individual words is also determined by a variety of intonational features, the most relevant of which for this study is the phrase-initial rise that marks the beginning of an accentual phrase. The interaction of the falling pitch accent and the phrase-initial rise is illustrated in the disyllabic minimal triplets in **Figure 1**, where the blue line above each word indicates a stylized F0 contour (in reality, there will be some interruptions in the F0 tracks due to the lack of voicing in /∫ /). The contrast between the three words is fully visible when they are followed by another word or morpheme. The unaccented /ha∫ i/ ‘edge’ shows no rapid pitch fall (**Figure 1a**), but the initially accented /há∫ i/ ‘chopsticks’ has a pitch fall between the first and second syllable (**Figure 1b**) and the finally accented /ha∫ í/ ‘bridge’ has a fall extending from the final syllable onto the following nominative marker (**Figure 1c**). The contrast between the unaccented /ha∫ i/ ‘edge’ and the finally accented /ha∫ í/ ‘bridge,’ however, is not observable when there is no following word or morpheme within the phrase (cf. **Figures 1d,f**). Furthermore, the rising pitch pattern shown in those two words disappears when they are not in phrase-initial position (**Figures 1g,i**), as the rise is a feature that marks the beginning of an accentual phrase. In contrast, the initially accented /há∫ i/ ‘chopsticks’ is consistently marked by a falling contour.

**Figure 1** also shows an autosegmental analysis of the structure underlying these pitch contours, based on the Pierrehumbert–Beckman model of Japanese prosodic structure (Beckman and Pierrehumbert, 1986; Pierrehumbert and Beckman, 1988) and its successor, the J-Tobi model (Venditti, 2005). Under this framework, the lexically defined pitch fall is seen as a realization of H^∗^L, a sequence of high (H) and low (L) tones. The H^∗^ portion of this tone combination docks on to the syllable that is lexically marked as accented. The onset of an accentual phrase is marked by a delimitative low tone (%L), followed by a high phrasal tone (H-), unless the realization of the latter is preempted by the presence of the lexical H^∗^. Captured in this analysis is the composite nature of the pitch patterns exhibited by these words in different contexts, which can be understood as combinations of two types of basic tones (H and L) assigned at different levels (i.e., words and phrases).^1^

**FIGURE 2:**
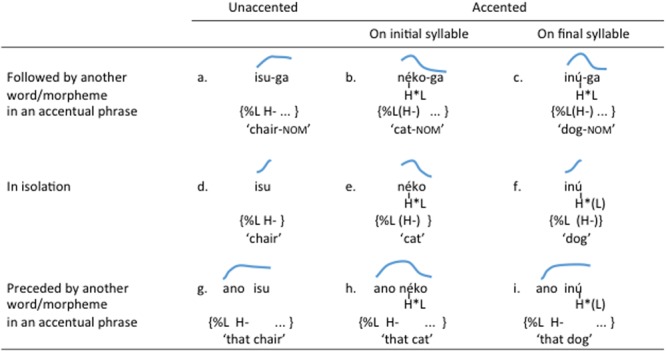
Three segmentally different Japanese words contrasting in pitch accent. Blue lines are styllized F0 contours. In **(a–c)**, *isu, neko*, or *inu* is followed by a nominative marker /ga/. In **(d–f)**, they are the only word in an accentual phrase (and therefore, phrase-initial). In **(g–i)**, they are not the initial word in an accentual phrase. Tonal analysis is given below each item.

While the interaction of lexical and non-lexical (intonational) pitch in Japanese words may be revealed unambiguously in such segmentally identical words, most words that a learner encounters do not come in minimal tonal pairs or triplets. Rather, words with different pitch profiles are typically also segmentally different, as illustrated in **Figure 2**. Given this type of input, how does a learner of Tokyo Japanese go about teasing apart the lexical and non-lexical components of pitch patterns? In particular, when do they understand that the variable pitch patterns associated with the unaccented /isu/ ‘chair’ (**Figures 2a,d,g**) and finally accented /inu/ ‘dog’ (**Figures 2c,f,i**) lexically mark those words in contrast with the falling pitch contour of the initially accented /neko/ ‘cat’ (**Figures 2b,e,h**)? How do they encode that information in their lexical knowledge of /isu/ and /inu/? Do they use pitch patterns to recognize those words even though they can be sufficiently identified on the basis of their segmental composition?

It is still not clear whether these aspects of the pitch accent phonology deter Japanese-learning infants from identifying the lexically relevant pitch contrasts. There is evidence that Japanese infants develop early sensitivity to the acoustic differences involved in the contrasts. As early as 4 months, they are capable of discriminating the falling vs. rising difference manifested in isolated words such as /há∫ i/ (‘chopsticks’) (**Figure 1d**) and /ha∫ í/ (‘bridge’) (**Figure 1f**) (Sato et al., 2010). By 10 months, they begin to show left-hemispheric dominance in processing the same pitch contrast embedded in words, but not when the contrast is presented in pure tones, suggesting that their perception of pitch contours becomes specialized for linguistic processing between 4 and 10 months (Sato et al., 2010). In contrast, there is scant empirical information as to when pitch contrasts become lexically incorporated in Japanese learners. Studies based on production data show that 15- to 24-month-olds consistently produce a falling contour for isolated initially accented words such as /neko/ (‘cat’) (**Figure 2d**), but vary in their extent to which they can produce a rising contour for isolated words with no or a non-initial accent such as /inu/ (‘dog’) (**Figure 2f**) (Hallé et al., 1991; Ota, 2003). This could be interpreted as evidence that Japanese-learning infants of this age have identified and learned the lexical falling pitch pattern but not the phrase-initial rise. However, a failure to produce a rising pitch contour may also be due to the additional articulatory effort required to produce a pitch rise compared to a pitch fall (Snow, 1998). The existing literature, therefore, fails to answer the question of how learning lexical contrasts in a lexical pitch accent language compares to the development of tone or non-tone languages.

### Purpose of the Current Study

Previous work indicates that learners of tone languages (e.g., Mandarin) can use pitch in recognition of familiar words by 11 months and in novel word learning by 18 months. Learners of non-tone languages (e.g., English) before 18 months are also able to lexically encode pitch variations. This suggests that regardless of what lexical role pitch plays in the target language, infants before 18 months are capable of extracting the relevant pitch patterns associated with lexical input and encode them in their lexicon. Can this ability also be exploited in learning a pitch accent system such as Japanese despite the complexities described above, which might obscure the lexically relevant patterns? This should be possible if Japanese infants are tracking the whole range of pitch patterns that are associated with individual words. For example, they may store exemplars of the final-accented word /inu/ ‘dog’ with a rising contour (**Figures 2c,f**) and a flat pattern (**Figure 2i**), allowing them to recognize both patterns as familiar forms even before they master the role of the accentual phrase. From that point of view, we expect Japanese-learning infants before 18 months of age to be able to differentiate words on the basis of pitch variations that correspond to a lexical contrast (i.e., rising vs. falling contour).

In this study, we investigated this question by experimentally testing the extent to which modifications in pitch contour can affect recognition of words that Japanese infants are likely to be familiar with. Words that infants frequently hear in their linguistic input are subject to natural variation in pitch including, crucially, the phrase-initial intonational marking that makes the rising pitch a variable feature. Testing recognition of familiar words, therefore, allows us to see whether infants overcome such input variability in integrating pitch information into lexical representations. To this end, we employed the mispronunciation paradigm (Swingley and Aslin, 2000) to test Japanese-learning 17-month-olds and examined their recognition of phrase-initial words with no accent or a final lexical pitch accent (e.g., /inu/ ‘dog’ in **Figure 2c**) when we imposed a falling pitch contour on those words, making them (incorrectly) initially accented. If, by this age, Japanese infants have developed understanding of the lexical function of this pitch contrast, they should show better recognition of the test words with the correct (i.e., rising) contour compared to the incorrect (i.e., falling) contour.

## Materials and Methods

### Overview

The participants in the experiment were 18 17-month-olds learning Tokyo Japanese. In each trial during the experiment, the infants saw two pictures on the monitor, accompanied by a recorded sentence naming one of the visual objects. In some trials, the target picture was named with the ‘correct’ pitch contour on the test word, while in some trials, it was named with an ‘incorrect’ pitch contour. There were also some filler trials in which a cartoon character familiar to many Japanese children was named with the correct pronunciation. Infants’ fixation to the visual objects was recorded using an eye-tracker.

### Participants

The 18 participants ranged in age from 17 months to 4 days (520 days) to 17 months and 30 days (546 days), with a mean of 17 months and 20 days (537 days). Half of them were female. One additional participant was tested but not included in the analysis due to eye-tracking failure caused by fussiness. All infants were born full-term and had no known history of ear infection or hearing problems. All infants also had parents who grew up in the vicinity of Tokyo, where the lexical accent of the test words followed the patterns illustrated in **Figures 1, 2**. None of them was reported having regular exposure to languages other than Japanese. Written informed consent was obtained from the parents of the participants.

### Materials

#### Auditory Stimuli

**FIGURE 3:**
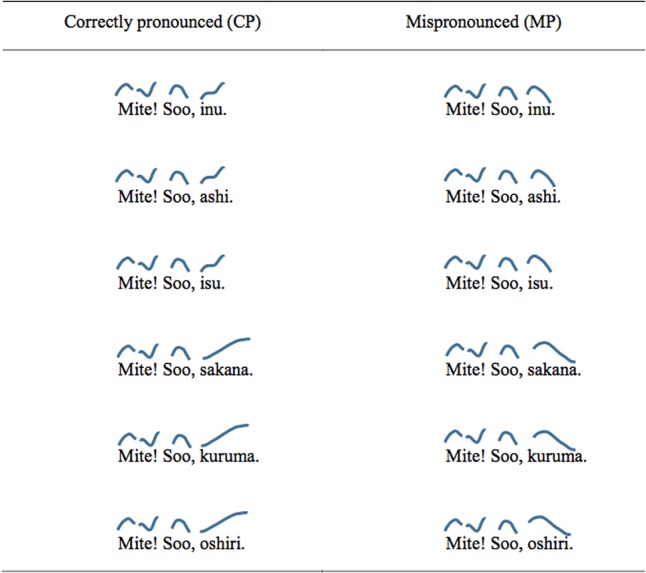
Examples of carrier passages with the experimental items. Each passage was also followed by *kawaii ne, omoshiroi ne*, or *wakaru ka na*. Lines above the words are stylized representations of the pitch contour.

**FIGURE 4:**
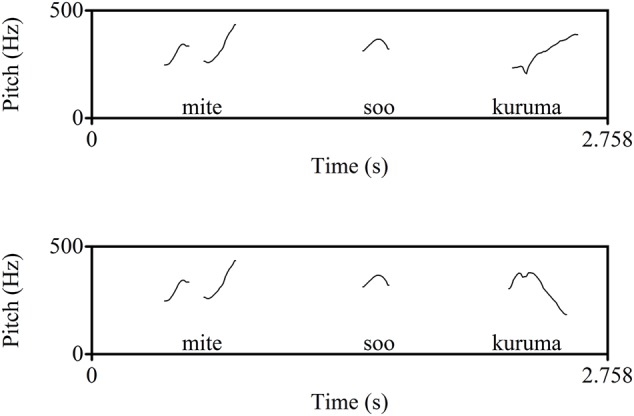
F0 extraction of the carrier phrase with the test word *kuruma* for the CP condition (top) and the MP condition (bottom).

The test words comprised three sets of words: Experimental words produced with the expected pitch contour (‘Correctly Pronounced’ or ‘CP’ words), experimental words produced with an unexpected pitch contour (‘Mispronounced’ or ‘MP’ words), and filler words, which were names of cartoon characters, always produced with the correct pitch contour. The CP and MP versions of the experimental words were created from 3 disyllabic words (*inu* ‘dog’, *isu* ‘chair’ and *ashi* ‘leg’) and 3 trisyllabic words (*sakana* ‘fish,’ *kuruma* ‘car,’ and *oshiri* ‘bottom/buttocks’). They either had a lexical pitch accent on the final syllable (*inu, ashi*) or no pitch accent (the rest). Each of these words was embedded in the carrier passage *Mite! Soo*, [target] (‘Look! Yes, [target]’), and said in a way such that it formed an independent prosodic phrase at the end of the sentence. The CP version had a rising pitch contour, as expected for a phrase-initial word without initial lexical accent. The MP version had a falling pitch contour, which, (incorrectly) signals an initial pitch accent. Each carrier passage was followed by one of the additional phrases, *kawaii ne* (‘Isn’t that cute.’), *omoshiroi ne* (‘Isn’t that interesting.’) or *wakatta ka na* (‘Did you get it?’). These phrases were added simply to break the monotony of the carrier passages without affecting the interpretation of the critical component of the stimuli. Combination of the additional phrase with the main part of the carrier passage was fully crossed. **Figure 3** shows schematic representations of these experimental stimuli, and **Figure 4** gives actual F0 extractions from the CP and MP versions of the recordings for *kuruma* ‘car.’

The filler words were *Ampamman, Doraemon, Mikkii* (Mickey Mouse) and *Puu-san* (Winnie the Pooh). The first two occurred in the carrier passage *Are? ___ da, Omoshiroi ne* (‘Hm? That’s ___. Isn’t that interesting.’) and the other two in the carrier passage *A! ___ da yo. Kawaii ne.* (‘Oh! There’s ___. Isn’t that cute.’).

The stimuli were read by a female native speaker of Japanese, using infant-directed speech, and digitally recorded in a sound-proof room at a sampling rate of 44.1 kHz (16 bit). Sound files were spliced so that the same recording of the carrier passages was used across experimental words. They were also normalized for amplitude.

#### Visual Stimuli

The visual stimuli were colored illustrations of the objects and characters corresponding to the experimental and filler words: a dog, a chair, a leg, a fish, a car, buttocks, Ampamman, Doraemon, Mickey Mouse, and Winnie the Pooh. The images were yoked in pairs based on their semantic characteristics: dog with fish, leg with buttocks, chair with car, Ampamman with Doraemon, and Mickey Mouse with Winnie the Pooh. They were presented side by side against a black background on a 24-inch wide-screen monitor (1920 pixels × 1200 pixels, approximately 57.3 cm × 45.0 cm). On the screen, the pictures were approximately 480 pixels × 360 pixels in diameter and separated by about 480 pixels.

### Procedure

The experiment was conducted in a dimly lit sound-proof room. Infants sat on their parent’s lap, approximately 60 cm away from the stimulus-presenting monitor. Parents listened to masking music played through a headset so that they could not hear the auditory stimuli, and were also asked to look down to prevent their eyes from being targeted by the tracking device. The experiment was monitored by a researcher, who sat in a control area outside the room and watched the procedure through a closed-circuit TV monitor. Stimulus presentation was controlled by the E-Prime 2.0 software (Psychology Software Tools, Pittsburgh, PA, United States). Auditory stimuli were played through loudspeakers placed below the TV monitor. Eye-gaze data from the infants were collected using a Tobii T60XL eye-tracking system.

Before the experimental trials, a five-point calibration routine was run in order to calibrate the eye-tracker to the infant’s eyes. The experimental trials consisted of 12 test trials and 4 filler trials, for a total of 16 trials. Each trial was 8 s long, and began with the presentation of two images appearing side by side at the vertical center of the screen. The images simultaneously moved at a steady pace toward the top of the screen, then to the bottom and back to the center at the end of the trial. The carrier passage began 2 s after the beginning of the trial. The onset of the test word (both experimental words and the fillers) occurred at 5 s. Between the trials, an animated sequence of a rotating smiley face was played. When the infant’s gaze was fixated to the center of the screen, the experimenter started the following trial.

Four stimulus sets were used, each with two blocks of presentation. The second and fourth stimuli sets reversed the block order of the first and third. The third and fourth sets were left-right reflection of the first and second. Each of the six experimental words was tested once in each block, under the CP condition in one block and under the MP condition in the other. Each of the four filler words was tested once in each experiment, in either the first or second block. Each picture served twice as the target (on the right in one block, and on the left in the other) and twice as the distractor (also on the right in one block, and on the left in the other). Presentation order was randomized within block.

## Results

If, by 17 months, Japanese infants have learned that disyllabic words without an initial pitch accent must not have a falling pitch contour, they should be more accurate or faster at fixating on the target image in CP trials than in MP trials. If their understanding of lexical pitch accent is robust enough, we expect to find this effect throughout the experimental trials. However, previous work on early lexical representation using a similar paradigm found that mispronunciation effects sometimes diminish over the course of the experiment (Vihman et al., 2004). This occurs presumably because infants begin to accept the mispronounced versions of the familiar words in later trials when the lexical encoding of the critical contrasts is fragile. We therefore included trial order (i.e., first vs. second block) as a factor in our analysis.

**FIGURE 5:**
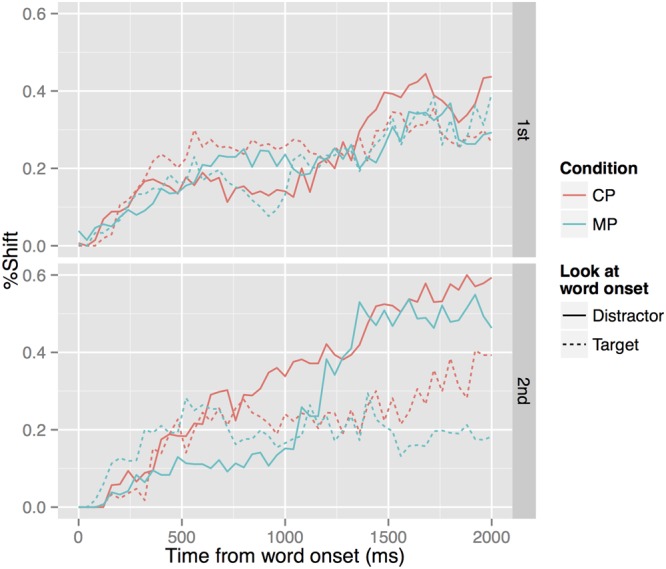
Onset-contingent eye-movement plots for the first block (top) and the second block (bottom). Solid lines track the movement for trials where the participants were looking at the distractor object at the onset of the test word, and dotted lines for trials where they were looking at the target object at the word onset. The *y*-axis shows the proportion of shifts (i.e., the proportion of looks to the opposite object).

The analysis was carried out using onset-contingent eye-movement data, which are summarized in **Figure 5**. These graphs display the time course of eye movement from the temporal onset of the test word, separately for the first block (top panel) and the second block (bottom panel). Within each panel, trials are aggregated into different lines depending on the condition (CP vs. MP) and the object at which the infant was looking at the word onset (target vs. distractor). For the purpose of the analysis, we call the object that matches the test word segmentally the ‘target’ picture whether the pitch contour was correct or not. For example, the picture of the dog was the target for both /inu/ (CP) and /inu/ (MP) and the yoked picture of the fish was the distractor for those words. Conversely, the picture of the fish was the target for both /sakana/ (CP) and /sakana/ (MP). The *y*-axis shows the proportion of fixation shifts to the opposite visual object for each 40 ms from the word onset. In the case of target-initial trials, this is the proportion of looks to the distractor over the sum of target and distractor looks. In the case of distractor-initial trials, this is the proportion of looks to the target over the sum of target and distractor looks. The analysis did not include trials in which the infant was looking neither at the target object or the distractor at the onset of the test word, which accounted for 22.4% of the data.

Following previous literature on fixation latency of this age range, we chose to analyze the gaze data from 360 to 2000 ms after word onset (Fernald et al., 1998), and modeled the time course of fixation shifts using growth-curve analysis (Mirman, 2014). All modeling was carried out using the lme4 package (Bates et al., 2015) on R. Time bins of 40 ms were created from the word onset and transformed to second-order orthogonal polynomial values to avoid correlations between time terms. We first ran two base models, one with the linear time term and one with both the linear and quadratic time terms. Both models also included by-participant random intercepts and slopes. As comparison of these models showed that adding a quadratic term to a linear-only model improved the model fit [χ^2^(4) = 42.71, *p* < 0.001], all subsequent models were built with linear and quadratic time terms (both with polynomial values). Next we ran an omnibus analysis using the two time terms (Time and Time^2^), Onset Look (Target vs. Distractor), Condition (MP vs. CP) and Block (1st vs. 2nd) as fixed effects (including their interactions), as well as participant random effects on both Time and Time^2^, and participant-by-condition random effects on both Time and Time^2^. This analysis yielded significant 4-way, 3-way and 2-way interactions involving Block and the other fixed effects (see **Table 1** for full results).

**Table 1:** Summary of the omnibus growth-curve model.

Effects	Estimate	*SE*	df	*t*	*p*
(Intercept)	0.230	0.042	19	5.486	<0.001^∗∗∗^
Time	0.461	0.133	32	3.457	0.002^∗∗^
Time^2^	0.154	0.093	75	1.662	0.100
Block	0.012	0.062	20	0.824	0.844
Condition	0.026	0.046	21	0.574	0.572
Onset Look	–0.028	0.056	20	0.510	0.619
Time × Block	–0.490	0.103	4806	4.729	<0.001^∗∗∗^
Time^2^× Block	–0.264	0.103	4798	2.566	0.010^∗^
Time × Condition	–0.212	0.100	4797	2.113	0.034^∗^
Time^2^× Condition	–0.152	0.100	4790	1.531	0.126
Time × Onset Look	–0.101	0.100	4797	1.001	0.317
Time^2^× Onset Look	–0.203	0.101	4785	2.018	0.044^∗^
Block × Condition	0.033	0.024	4698	1.405	0.160
Block × Onset Look	0.138	0.025	4740	5.475	0.037^∗^
Condition × Onset Look	0.076	0.022	4799	3.394	<0.001^∗∗∗^
Time × Block × Condition	0.561	0.144	4802	3.886	<0.001^∗∗∗^
Time^2^× Block × Condition	0.386	0.141	4775	2.695	0.007^∗∗^
Time × Block × Onset Look	1.278	0.147	4806	8.664	<0.001^∗∗∗^
Time^2^× Block × Onset Look	0.269	0.147	4763	1.834	0.067
Time × Condition × Onset Look	0.564	0.141	4793	4.012	<0.001^∗∗∗^
Time^2^× Condition × Onset Look	0.314	0.140	4796	2.236	0.025^∗^
Block × Condition × Onset Look	–0.068	0.034	4794	2.035	0.042^∗^
Time × Block × Condition × Onset Look	–1.016	0.204	4801	4.979	<0.001^∗∗∗^
Time^2^ × Block × Condition × Onset Look	–0.570	0.203	4759	2.809	0.005^∗∗^

*Parameter estimates are for CP relative to the MP (Condition), Block 2 relative to Block 1, and distractor-initial relative to target-initial (Onset Look). ^∗^*p* < 0.05, ^∗∗^*p* < 0.01, ^∗∗∗^*p* < 0.001.*

In order to tease apart these interactions, we proceeded to build separate models for the two blocks. In these models, Block and its interactions with other factors were removed. The results for Block 1 are given in **Table 2**. There were significant interactions between Onset Look and Condition on Time and Time^2^, with the linear term indicating a generally faster overall shift from the distractor to the target for the CP condition relative to the MP condition (Estimate = 0.624, *SE* = 0.139, *p* < 0.001) and the quadratic term indicating more acceleration in the distractor-to-target shift for the CP condition relative to the MP condition (Estimate = 0.273, *SE* = 0.139, *p* = 0.049). There was also a significant interaction between Onset Look and Condition in reflection of an overall higher level of distractor-to-target shift in the CP condition than the MP condition (Estimate = 0.048, *SE* = 0.023, *p* = 0.037). In addition, there was an effect of Condition on Time, suggesting that the overall speed of shift was slower for the CP condition relative to the MP condition (discounting the Condition × Onset Look interaction mentioned above) (Estimate = -0.256, *SE* = 0.100, *p* = 0.010). However, there were no interactions of Onset Look and the time terms. These results indicate that the infants were more likely to shift their gaze from the distractor to the target object and did so faster than target-to-distractor shifts but only in the CP condition. In short, their distractor-to-target response was contingent on hearing the target word with the correct pitch contour.

**Table 2:** Summary of the growth-curve model for Block 1.

Effects	Estimate	*SE*	df	*t*	*p*
(Intercept)	0.239	0.047	14.2	5.136	<0.001^∗∗∗^
Time	0.496	0.155	24.9	3.210	0.004^∗∗^
Time^2^	0.125	0.107	37.0	1.164	0.252
Condition	0.042	0.051	19.1	0.824	0.420
Onset Look	–0.033	0.062	18.9	0.541	0.595
Time × Condition	–0.256	0.100	2483.3	2.567	0.010^∗^
Time^2^× Condition	–0.101	0.099	2456.2	1.027	0.305
Time × Onset Look	–0.124	0.100	2482.9	1.235	0.217
Time^2^× Onset Look	–0.145	0.100	2456.9	1.450	0.147
Condition × Onset Look	0.048	0.023	2495.8	2.082	0.037^∗^
Time × Condition × Onset Look	0.624	0.139	2477.8	4.481	<0.001^∗∗∗^
Time^2^× Condition × Onset Look	0.273	0.139	2476.3	1.968	0.049^∗^

*Parameter estimates are for CP relative to the MP (Condition) and distractor-initial relative to target-initial (Onset Look). ^∗^*p* < 0.05, ^∗∗^*p* < 0.01, ^∗∗∗^*p* < 0.001.*

The results for Block 2 are given in **Table 3**. There was a significant interaction between Onset Look and Condition on Time, indicating a generally slower overall shift from the distractor to the target for the CP condition relative to the MP condition (Estimate = -0.439, *SE* = 0.115, *p* < 0.001). However, there was again a significant interaction between Onset Look and Condition, indicating an overall higher level of distractor-to-target shift in the CP condition than the MP condition (Estimate = 0.144, *SE* = 0.024, *p* < 0.001). These outcomes are likely due to the changing rates in the competitor-to-target shift in the MP condition, which showed little movement up to about 1000 ms post-naming, but a rapid increase toward the 1400 ms point, after which it plateaued. In comparison, the temporal change in the CP condition was more monotonic. Importantly, there was also a significant effect of Onset Look on Time, showing that the distractor-to-target shift was faster than the target-to-distractor shift across conditions (Estimate = 1.200, *SE* = 0.086, *p* < 0.001). In addition, there was an effect of Condition on Time, this time suggesting that the overall speed of shift was faster for the CP condition relative to the MP condition (discounting the Condition × Onset Look interaction mentioned above) (Estimate = 0.371, *SE* = 0.082, *p* < 0.001). These results indicate that, unlike in Block 1, infants were more likely to shift their gaze from the distractor to the target in both the MP and CP conditions, although the onset of the response was delayed in the MP condition compared to the CP condition.

**Table 3:** Summary of the growth-curve model for Block 2.

Effects	Estimate	*SE*	df	*t*	*p*
(Intercept)	0.187	0.055	12.5	3.371	0.005^∗∗^
Time	–0.135	0.168	21.5	0.805	0.430
Time^2^	–0.030	0.081	46.3	0.369	0.714
Condition	0.064	0.087	15.8	0.730	0.476
Onset Look	0.018	0.083	18.5	0.214	0.833
Time × Condition	0.371	0.082	2278.3	4.552	<0.001^∗∗∗^
Time^2^ × Condition	0.115	0.080	2209.3	1.427	0.154
Time × Onset Look	1.200	0.086	2290.0	13.952	<0.001^∗∗∗^
Time^2^ × Onset Look	–0.025	0.085	2058.1	0.292	0.770
Condition × Onset Look	0.144	0.024	1992.0	6.071	<0.001^∗∗∗^
Time × Condition × Onset Look	–0.439	0.115	2277.4	3.810	<0.001^∗∗∗^
Time^2^ × Condition × Onset Look	–0.104	0.114	2227.2	0.912	0.362

*Parameter estimates are for CP relative to the MP (Condition) and distractor-initial relative to target-initial (Onset Look). ^∗^*p* < 0.05, ^∗∗^*p* < 0.01, ^∗∗∗^*p* < 0.001.*

The overall level of distractor-to-target shift was higher in the second block than in the first. In Block 1, the proportion of distractor-to-target shift did not reach 50% even between 1500 and 2000 ms in either the CP (mean = 39.1%) or MP (mean = 31.0%) condition. In Block 2, the mean shift proportion between 1500 and 2000 ms was 55.5% for the CP condition and 49.6% for the MP condition, although the difference in distractor-to-target shift between the two conditions was not statistically significant.

## Discussion

In this study, we examined whether 17-month Japanese-learning infants understand the contrastive nature of the pitch patterns in familiar words. Our focus was on phrase-initial unaccented and finally accented disyllabic words such as /isu/ ‘chair’ and /inú/ ‘dog,’ which have a rising pitch pattern as opposed to the falling pitch pattern found in initially accented disyllabic words such as /néko/ ‘cat.’ A point of particular interest was that the pitch rise is not a unique lexical marker of the unaccented and finally accented words, and the lexical contrast needs to be understood as a *lack* of the falling pitch contour that unambiguously defines initially accented words. We predicted that Japanese learning infants should be able to learn this contrast by exploiting the type of ability exhibited by both tone and non-tone language learners of similar ages to encode pitch information in lexical representation. The results of our experiment present some evidence that 17-month-olds indeed utilize pitch information in recognizing words such as /isu/ and /inu/. In early trials, infants were faster in shifting their gaze from the distractor object to the target object when the test word correctly had a rising pitch contour than when it incorrectly had a falling contour. This part of the results indicates that despite the variable realizations of the pitch contours, Japanese-learning infants by this age have internalized some information about one of the possible pitch patterns (i.e., the rising contour) of these words to the extent that the online recognition process was facilitated by pitch-matching.

This difference between the correct and incorrect conditions, however, did not persist into later trials, during which infants showed faster distractor-to-target shifts than target-to-distractor shifts both when the test words were ‘mispronounced’ with a falling contour as well as when they were correctly pronounced with a rising contour. Although the pitch-mismatched words caused a slight delay in the onset of the distractor-to-target shift, they induced as much target object fixation as did the pitch-matched words within 2 s. The willingness infants exhibited in accepting such mappings suggests that the lexical encoding of pitch information is not firmly established enough to reject a mismatch in pitch in later trials. This outcome is similar to that from one of the experiments conducted by Vihman et al. (2004) in which they tested 11-month English-learning infants on their auditory recognition of familiar words (e.g., *baby*) and mis-stressed words (e.g., *ba’by*) compared to rare words that are assumed to be unfamiliar (e.g, *bridle*). Tests using the head-turn preference paradigm showed no difference in the preference for mis-stressed words vs. rare words during the first half of the experiment, indicating that recognition of familiar words was blocked by the incorrect placement of stress. However, mis-stressed words were significantly preferred over rare words in the second half, suggesting that after exposure to examples such as *ba’by*, the infants began to regard the stress-mismatched words as familiar words. The emergent tendency to accept the pitch-mismatched words in our experiment might have been induced further by the nature of the task, which involved visual stimuli presented in pairs. In a visual world paradigm, participants’ processing of prosodic information can be guided incrementally by the contextual expectations signaled by the visual stimuli (Kurumada et al., 2014). In the case of the current experiment, once the infants register, for example, the fact that there is a picture of a dog (/inu/) as well as of a fish (/sakana/) on the screen, they are more likely to look toward the dog upon hearing the pitch-mismatched /inu/, simply because of its better segmental match with one of the options presented. The extent to which such expectation effects might have affected the outcome of our study can be gauged by testing the same linguistic stimuli using the wordform-only design employed in Vihman et al. (2004) and several other studies on the lexical representation of familiar words in infants (e.g., Hallé and de Boysson-Bardies, 1994, 1996; Swingley, 2005; Vihman and Majorano, 2017).

These methodological considerations notwithstanding, the results here indicate that 17-month-olds are still in a nascent state when it comes to their grasp of the lexical import of the rise/fall contrast in Japanese. This timing of development seems rather protracted given the evidence that Japanese infants can perceptually discriminate the same contrast as early as 4 months (Sato et al., 2010), and both Mandarin and English infants of similar or younger ages are capable of encoding a rise/fall contrast in novel words through brief lab exposure (Singh et al., 2014; Hay et al., 2015). As foreshadowed in the Introduction, such a delay is mostly likely caused by the variable realization of pitch patterns introduced by the interaction of lexical and non-lexical factors in Japanese pitch phonology. A Japanese infant who hears the word /inu/ ‘dog’ sometimes with a pitch rise and sometimes with a flat pitch pattern may conclude (correctly) that the rise/plateau alternation is lexically irrelevant, but may fail to notice — precisely because of this variability — that the contrast between a rise or a plateau, on the one hand, and a fall, on the other, is lexically relevant. Note that such input variability is not a feature of experiments that demonstrate successful mapping of lexical tones with novel words by both Mandarin and English infants (e.g., Singh et al., 2014; Hay et al., 2015), because in these studies, the stimuli are played consistently in one type of lexical tone during familiarization. Hence, the ability to lexically encode pitch from invariable exemplars does not guarantee successful extraction of lexically contrastive pitch patterns in the face of variable realizations. Further support for this interpretation comes from a finding reported by Shi et al. (2017) for familiar word recognition by Mandarin learners. As in our study, Shi et al. (2017) used the mispronunciation paradigm with visual references, and tested whether monolingual Mandarin learners between 19 and 26 months would recognize familiar words when an incorrect tone was assigned. Their participants detected mispronunciations involving Tone 2 (rising tone) and Tone 4 (falling tone) or Tone 3 (dipping tone) and Tone 4, demonstrating that they have internalized these tonal contrasts in their lexical knowledge. However, the same individuals did not detect mispronunciations involving the contrast between Tones 2 and 3. Shi et al. (2017) reject perceptual confusion as a source of this failure because younger Mandarin learners are capable of discriminating Tones 2 and 3. Instead, they attribute the lack of mispronunciation effects for Tone 2/3 to the variable realization of Tone 2. As discussed in the Section “Introduction,” in Mandarin, Tone 3 (dipping tone) is realized as Tone 2 (rising tone) when followed by another Tone 3. Mandarin infants, therefore, are exposed to words whose pitch pattern alternates between a dipping contour and a rising contour, potentially leading them to inaccurately encode both dipping and rising patterns as contextually constant representations of Tone 3 words. Variability is also a potential factor behind the apparently late pitch phonology development in Limburgian (Ramachers et al., 2017). Like Japanese, Limburgian has one type of tonal contrast that is lexically assigned to a syllable in each word, but its pitch realization varies dramatically across intonational contexts (e.g., declarative, interrogative, and continuation) (Gussenhoven and van der Vliet, 1999). Ramachers et al. (2017) trained 2.5- to 4-year-olds on novel word-object associations and subsequently tested their word recognition using a mispronunciation design. Their Limburgian learners fixated on the target object even when they heard a pitch-mismatched version of the novel word, suggesting that the pitch differences were not treated as a lexical contrast. It is difficult to compare this result with that of our study, given the differences in age, methodology (in particular, the use of novel words as opposed to familiar words), and linguistic environment (the Limburgian toddlers were also heavily exposed to Dutch)^2^. Yet, they are both consistent with the notion that the task of learning pitch contrasts could be made arduous when their realizations are subject to variability due to non-lexical factors.

A slightly different point that is nevertheless pertinent to the issue of variability is the phonological contexts in which words tend to appear in the learner’s speech input. If infants hear words such as /inu/ ‘dog’ and /isu/ ‘chair’ predominantly in single-word utterances (as in **Figures 2d,f**), the pitch contrast against initially accented words such as /neko/ (**Figure 2e**) will be more noticeable because it will be realized as a difference between a rising and a falling contour in the large majority of the cases, and because the size of the phonological material over which the critical contrast is expressed is small (i.e., a single word, which is also the entire phrase and utterance). This means that the question as to how easily learners can unravel the prosodic phonology that underlies the observable pitch patterns in the language is dependent not only on the nature of the system (e.g., lexical tone, lexical pitch, intonation) but also on how the critical contrasts are made more apparent by the distributional relationship between words, phrases and utterances in the ambient input. This principle may also apply to the development of non-tone languages. For example, Frota et al. (2014) demonstrated that both 5–6-month-olds and 8–9-month-olds learning European Portuguese (EP) could discriminate the intonational patterns associated with the declaratives (HL^∗^ L%) vs. yes-no questions (HL^∗^ LH%) of the language. In Soderstrom et al. (2011), however, infants between 4 and 24 months failed to classify the declarative vs. yes-no question patterns in English, albeit showing a preference for yes-no questions. One likely explanation for these different results is that the stimuli in Frota et al. (2014) were single-word utterances consisting of disyllabic words whereas those in Soderstrom et al. (2011) were multi-word utterances with the critical prosodic differences marked mostly at the end of the utterance. Furthermore, there is an indication that the proportion of single-word intonational phrases in infant-directed speech is much higher in EP than in English (Frota et al., 2014). For these reasons, the intonational contrast between declaratives and yes-no questions may be more tractable in EP than in English. An analysis of infant-directed Japanese may reveal that Japanese does not lean heavily toward single-word prosodic phrases as EP does, thus showing less scaffolding for the learner in this respect.

There are other factors that could pose a challenge to acquiring a lexical pitch accent system, especially in contrast to a lexical tone system. First, pitch has a lower functional load in pitch accent languages than in many tone languages. Because a lexical pitch accent system typically has only one type of lexically significant pitch pattern (e.g., a fall), which is also assigned only up to one syllable per word, it has far fewer minimal pairs that rely solely on pitch differences in comparison to tone languages. As such, the function that pitch plays in lexical contrasts may be less readily noticeable by the learner. Second, there may be a difference in perceptual salience between a pitch accent and lexical tones. Lexical tones are typically realized within a syllable, so the contour pattern is audible as a continuous sonorant unit. In contrast, single syllable realization of a lexical pitch accent can be limited to certain types of syllables (e.g., those that contain a long vowel or a sonorant coda in Japanese), and the contour of a pitch accent is otherwise interrupted by a syllable boundary. It is possible that learners find it more difficult to perceive pitch movements that are phonetically discontinuous. There may also be acoustic differences when similar pitch contours are compared between tone languages and lexical pitch languages. While the mean onset-to-offset F0 movements in our rising (232–388 Hz) and falling (375–184 Hz) pitch items are fairly comparable with, for example, Singh et al.’s (2014) Mandarin stimuli for rising/Tone 2 (221–346 Hz) and falling/Tone 4 (324–206 Hz), the F0 movements in the phrase-initial rise may be less pronounced in naturalistic infant-directed Japanese (Ota, 2003).

Our study examined only part of the knowledge 17-month-olds may have of the pitch accent system in Japanese. All the target words investigated here either had no lexical accent or an accent on the final syllable. Future studies should include testing of infants’ response to initially accented words mispronounced with a rising contour as opposed to the correct falling contour. We predict that 17-month-olds should display stronger sensitivity to this mismatch because initially accented words are consistently marked by a falling contour (cf. **Figures 2b,e,h**), making any deviation from the pattern straightforwardly anomalous. An equally important issue that has been left unexplored here is how the non-lexical (i.e., intonational) component of pitch patterns is acquired. This can be decomposed into two issues. First, infants must learn that pitch changes caused by non-lexical factors, such as phrasal boundaries, do not have lexical consequences. This question can be addressed by testing, for example, whether Japanese-learning infants recognize words with no or non-initial accent in phrase-initial as well as non-initial position (cf. **Figures 2g–i**), where the rising contour disappears. Second, infants must also learn that certain pitch patterns are required by sentence structure or meaning, rather than words. This can be examined by testing whether infants detect anomalies in utterances that lack a phrase-initial rise when one is expected (e.g., **Figures 2d,f–i**). If lexical encoding of invariable pitch patterns plays an important role in the initial phase of pitch development, we expect such intricacies of non-lexical pitch phonology to be acquired only after some amount of lexical information has accumulated in the learner, for it is only when the contribution of word-level prosody is understood that many aspects of intonational phonology become evident. In this regard, it is interesting to note that there is a consensus emerging from research on early speech production in non-tone languages, including Catalan, Dutch, English, and Spanish, that the timing of intonational development is linked not to sentence length but lexical knowledge (Chen and Fikkert, 2007; DePaolis et al., 2008; Prieto et al., 2012).

To summarize, 17-month-old Japanese infants have internalized some lexically relevant pitch information of familiar words, but the information does not withstand the pressure to segment-match a pitch-mismatch word. On the one hand, this means that by this age infants can extract lexically relevant pitch patterns in the face of variability introduced by non-lexical (intonational) factors. On the other hand, however, lexical knowledge of pitch contrast in 17-month-old Japanese infants does not appear to be on a par with that found in similar-aged Mandarin infants, at least where comparable pitch contour differences (i.e., rising vs. falling) are concerned. Further research will shed light on whether such differences reflect the developmental complexities involved in decoupling lexical and intonational features in pitch phonology. In this respect, examination of the development of pitch accent languages offers insights that complement those emerging from relatively well-researched systems such as lexical tone languages and non-tone languages. The current study constitutes a step toward a more comprehensive understanding of how non-segmental lexical contrasts develop during infancy.

## Ethics Statement

This study was carried out in accordance with the recommendations of British Psychological Society with written informed consent from all subjects. The protocol was approved by the School of Philosophy, Psychology and Language Sciences, University of Edinburgh.

## Author Contributions

MO designed the study, analyzed the data, and drafted the manuscript. NY prepared the experimental materials and set-up, collected and analyzed the data, and co-wrote the manuscript. RM supervised the project and co-wrote the manuscript.

## Conflict of Interest Statement

The authors declare that the research was conducted in the absence of any commercial or financial relationships that could be construed as a potential conflict of interest.

## Footnotes

1 These models of Japanese prosody also propose higher levels of structure that assign non-lexical tones (the ‘intermediate phrase’ and ‘utterance’ in Pierrehumber–Beckman, and the ‘intonation phrase’ in J-Tobi). These levels are not included in the discussion here as they do not have immediate bearing on our study.

2 Another source of complication is that pitch mispronunciation did not block word recognition in either their age-matched Dutch toddlers or adult Limburgian speakers.
